# Chemical Characteristics and Source Identification of PM_2.5_ in Industrial Complexes, Korea

**DOI:** 10.3390/toxics14020111

**Published:** 2026-01-23

**Authors:** Hyeok Jang, Shin-Young Park, Ji-Eun Moon, Young-Hyun Kim, Joong-Bo Kwon, Jae-Won Choi, Cheol-Min Lee

**Affiliations:** Department of Environmental and Chemical Engineering, Seokyeong University, 14, Seogyeong-ro, Seongbuk-ku, Seoul 12713, Republic of Korea; amer1can@skuniv.ac.kr (H.J.); tlsdud060900@skuniv.ac.kr (S.-Y.P.); mje0313@skuniv.ac.kr (J.-E.M.); rladudgus128@skuniv.ac.kr (Y.-H.K.); knt8888@skuniv.ac.kr (J.-B.K.); 2025714006@skuniv.ac.kr (J.-W.C.)

**Keywords:** particulate matter, atmospheric pollution, receptor model, emission source

## Abstract

The composition of air pollutants in industrial complexes differs from that of general urban areas, often containing more hazardous substances that pose significant health risks to both workers and residents nearby. In this study, PM_2.5_ and its 29 chemical components (eight ions, two carbon species, and 19 trace elements) were measured and analyzed at five monitoring sites adjacent to the Yeosu and Gwangyang industrial complexes from August 2020 to December 2024. Chemical characterization and source identification were conducted. The average PM_2.5_ concentration was 18.63 ± 9.71 μg/m^3^, with notably higher levels observed during winter and spring. A low correlation (R = 0.56) between elemental carbon (EC) and organic carbon (OC) suggests a dominance of secondary aerosols. The charge balance analysis of [NH_4_^+^] with [SO_4_^2−^], [NO_3_^−^], and [Cl^−^] showed slopes below the 1:1 line, indicating that NH_4_^+^ is capable of neutralizing these anions. Positive matrix factorization (PMF) identified eight contributing sources—biomass burning (10.4%), sea salt (11.8%), suspended particles (7.1%), industrial sources (4.6%), Asian dust (5.2%), steel industry (21.8%), secondary nitrate (16.4%), and secondary sulfate (22.7%). These findings provide valuable insights for the development of targeted mitigation strategies and the establishment of effective emission control policies in industrial regions.

## 1. Introduction

Particulate matter (PM) has significantly influenced air quality, visibility, public health, and socio-economic aspects throughout human history [1,2,3,4,5]. According to the World Health Organization (WHO), more than 99% of the global population is exposed to air pollution levels exceeding WHO air quality guidelines [6]. In particular, fine particulate matter (PM_2.5_, particulate matter with an aerodynamic diameter less than 2.5 μm) has been associated with cardiovascular diseases, respiratory illnesses, and premature mortality, raising significant public concerns [7,8,9,10,11]. Consequently, effective mitigation strategies for PM_2.5_ are essential.

Due to its small particle size, PM_2.5_ can adsorb various chemical species, including aldehydes such as formaldehyde (HCHO), volatile organic compounds (VOCs), heavy metals (HMs), and water-soluble inorganic ions (WSIIs) [12,13,14,15]. The chemical composition of PM_2.5_ varies depending on its emission sources [16,17,18]. In urban environments, vehicular emissions contribute to high concentrations of organic carbon (OC), elemental carbon (EC), and Mn [19,20,21,22]. In contrast, construction sites are characterized by elevated levels of ionic species such as Ca^2+^ and Mg^2+^, as well as elements such as Al, Si, Ti, and Fe [23,24]. Considering the implications of PM_2.5_ for air quality management, ongoing efforts have focused on characterizing its chemical composition and identifying emission sources [25,26].

Studies on source apportionment aim to quantify the contributions of individual emission sources to PM_2.5_ and its chemical constituents. Receptor models, which analyze PM_2.5_ composition from collected samples, are powerful tools for source identification [27,28,29]. Among these models, positive matrix factorization (PMF) is widely used globally as it enables source contribution estimation without requiring prior knowledge of emission source profiles [30,31]. The PMF model is particularly well-suited for application in South Korea [32] and is highly effective in analyzing both inorganic and metallic components [33].

The study area, encompassing Yeosu and Gwangyang, hosts large-scale petrochemical and steel industrial complexes. According to Hong et al. (2018), residents near these industrial zones have experienced air pollution-related exposure and reductions in lung function [34]. Additionally, Seo et al. (2014) reported high concentrations of odorous compounds, benzene, and carcinogens such as HCHO at industrial sites [35]. Furthermore, a health risk assessment of PM_2.5_ and heavy metals in Yeosu and Gwangyang revealed that specific age and gender groups exceeded acceptable risk levels, highlighting the urgent need for air quality management [36]. These findings underscore the necessity for source apportionment studies to guide effective air quality control measures. In this region, previous investigations have primarily focused on the spatial and temporal distribution of PM_2.5_, providing foundational information on concentration levels and their variability [37]. A comprehensive interpretation of these patterns and their implications for emission reduction strategies further requires the identification of dominant emission sources and an assessment of their quantitative contributions. This need is particularly pronounced in industrial complex areas, where diverse and overlapping emission activities complicate source–receptor relationships and call for receptor-based source apportionment approaches to support informed policy decision-making.

Therefore, this study investigates the spatial and chemical characteristics of PM_2.5_ and its components (eight ions, two carbon species, and 19 trace elements) based on sampling conducted from August 2020 to December 2024. PMF analysis was employed to estimate the contributions of various emission sources. The findings from this study will contribute to the development of targeted policies and strategies for mitigating localized air pollution and managing emission sources effectively.

## 2. Materials and Methods

### 2.1. Sampling Sites

**Figure 1:**
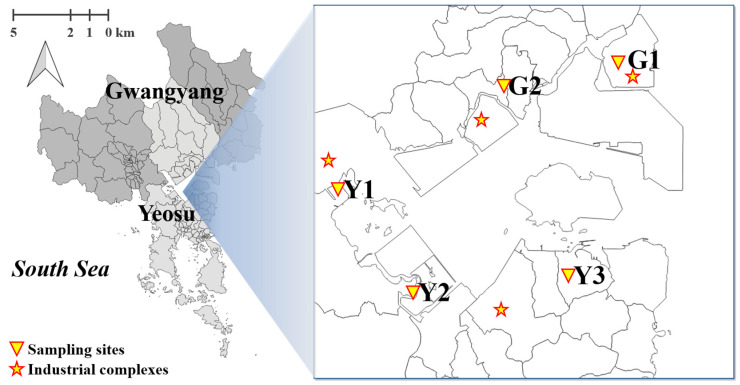
Sampling sites and industrial complexes in this study.

PM_2.5_ samples were collected from August 2020 to December 2024 in five residential areas near the Yeosu and Gwangyang industrial complexes. These locations were selected based on the distribution of emission sources and geographical characteristics to ensure direct exposure to industrial emissions (Figure 1). The monitoring instruments were installed in public facilities within the selected areas, ensuring the absence of obstructions such as buildings and trees and the availability of a stable power supply. Detailed information on site selection and measurement locations can be found in our previous study [37], and the specific characteristics of each monitoring site are presented in Table 1. Meteorological data measured at an Automatic Weather Station (AWS) operated by the Korea Meteorological Administration (KMA) and located near the study area were used to characterize local meteorological conditions. The AWS (34°51′02.01″ N, 127°42′23.67″ E) was situated in the vicinity of site Y3 and provided continuous measurements of wind direction, wind speed, temperature, and relative humidity [38]. The meteorological data obtained from this station are presented in Figure S1 and Table S1. Seasonal wind pattern analysis indicated that northerly winds were predominant during spring, whereas northeasterly winds were dominant during summer and autumn. In winter, the strongest wind speeds among the four seasons were observed, with prevailing northwesterly winds associated with the influence of the Siberian air mass. Overall, the seasonal variation in wind direction and strength suggests that atmospheric dispersion in the study area is influenced by air masses originating from multiple directions throughout the year.

### 2.2. PM_2.5_ Sampling

PM_2.5_ sampling was conducted twice a week (on Mondays and Wednesdays) during spring (March to May) and winter (December to February), when high particulate matter concentrations have been reported in South Korea. In summer (June to August) and autumn (September to November), sampling was conducted weekly on Mondays. PM_2.5_ samples were collected using a low-volume air sampler (PMS-204, APM Co., Ltd., Bucheon-si, Gyeonggi-do, Republic of Korea) following the US EPA FRM method at a flow rate of 16.7 L/min. Sampling commenced at 8:00 AM and continued for 24 h. The specifications of the PM_2.5_ measurement equipment used in this study are presented in Table S2.

PTFE filters (2.0 μm, Ø27 mm) were used for analyzing ionic and trace elements, while quartz fiber filters (Pure Quartz fiber, Ø47 mm) were employed for carbonaceous species analysis. Samples lost due to extreme weather conditions, such as typhoons and heavy rainfall, or equipment maintenance and repair (categorized as not applicable, N.A.) were excluded from the analysis. A total of 1775 PTFE filters and 1763 quartz fiber filters were collected over the entire study period. To minimize particle loss during transport, samples were stored in refrigerated containers below 4 °C with ice packs and protected with cushioning materials to prevent physical impact.

### 2.3. PM_2.5_ Chemical Components Analysis

In this study, we analyzed eight ionic species—three anions: Cl^−^, NO_3_^−^, SO_4_^2−^; five cations: Na^+^, NH_4_^+^, K^+^, Mg^2+^, Ca^2+^), two carbonaceous species (OC, EC), and 19 trace elements (Al, Ti, V, Mn, Fe, Ni, Co, Cu, Zn, As, Sr, Mo, Cd, Ba, Pb, P, S, Cr, and Si).

For ionic composition analysis, filters were stored in sterilized Petri dishes, sealed with parafilm, and refrigerated before analysis. Ethanol (200 μL) was added to each filter, which was then immersed in a beaker with the sampling side facing downward. Subsequently, 20 mL of ultrapure water (≥18 MΩ) was added, and the mixture was stirred at 120 rpm for 120 min to extract ionic species. The extracted solution was filtered using a 0.2 μm syringe filter and analyzed by ion chromatography (ICS-3000, Dionex, Sunnyvale, CA, USA) (Table S3).

For carbonaceous species analysis, filters were pre-treated by placing them on quartz plates without overlapping and heating them at over 550 °C for more than 4 h to remove moisture. The samples were then analyzed using a semi-continuous OC/EC analyzer (Model-5, Sunset Lab, Tigard, OR, USA) (Table S4). Trace element analysis was conducted using an energy-dispersive X-ray fluorescence (ED-XRF) analyzer (Epsilon 4, Malvern Panalytical, Worcestershire, UK) (Table S5).

### 2.4. Quality Assurance and Quality Control (QA/QC)

Quality assurance and quality control (QA/QC) procedures were conducted annually to ensure the accuracy of the measured components. The method detection limit (MDL), relative standard deviation (RSD), and calibration curve were evaluated. The calibration curve’s linearity coefficient (R^2^) was required to be above 0.99, and the RSD had to remain within 10%. Recalibration was performed if these criteria were not met. The QA/QC results confirmed that all components met the MDL and RSD criteria (Table S6), with R^2^ values exceeding 0.999.

### 2.5. Statistical Analysis

To assess the chemical characteristics of PM_2.5_ in the Yeosu and Gwangyang regions, descriptive statistical analysis and analysis of variance (ANOVA) were conducted to examine the concentration differences among monitoring sites. Values classified as not detected (N.D.) were excluded from the analysis to minimize statistical errors. Prior to conducting ANOVA, all data were normalized between 0 and 1 using z-score standardization, with a significance level set at 0.05.

The coefficient of divergence (COD) was calculated to evaluate the spatial distribution characteristics among the five monitoring sites. COD is a statistical metric used to assess spatial homogeneity and heterogeneity between two monitoring sites; values close to 1 indicate distinct emission sources, whereas values near 0 suggest similar sources [39,40,41]. Based on previous studies, a COD value exceeding 0.3 was considered indicative of differences in emission sources between sites [42,43,44]. The COD was calculated using Equation (1).

(1) $$COD=\sqrt{\frac{1}{n}\sum_{i=1}^{n}{\left(\frac{X_{ij}-X_{ik}}{X_{ij}+X_{ik}}\right)}^{2}}$$

Here, $X_{ij}$ represents the mean concentration of component i at measurement site j, where j and k denote two adjacent measurement sites, and n represents the number of measured components.

### 2.6. Source Identification

After identifying the chemical characteristics of PM_2.5_ in the Yeosu and Gwangyang areas, PMF 5.0 [45], one of the representative receptor models, was applied to determine the emission sources affecting air quality. PMF was first introduced by Paatero and Tapper (1994) [46] and is a quantitative method for estimating the source contributions to the mass concentrations of PM_2.5_ and its chemical components [47].

For the model execution, concentration files of chemical components and corresponding uncertainty files estimated based on concentration levels were used as input data. A speciated dataset can be represented as a data matrix X, where i denotes the number of sampling events and j represents the number of measured chemical species. The multivariate matrix model, PMF, is calculated using Equation (2).

(2) $$X_{ij}=\sum_{k=1}^{p}g_{ik}f_{kj}+e_{ij}$$

Here, p represents the number of identified factors, f denotes the species profile of each source, g refers to the amount of mass contributed by each factor to each sample, and e is the residual matrix for each sample/species.

The objective function Q, a critical parameter in the PMF model optimization, is minimized through an iterative algorithm within the model. It is calculated using Equation (3).

(3) $$Q=\sum_{i=1}^{n}\sum_{j=1}^{m}{\left[\frac{X_{ij}-\sum_{k=1}^{p}g_{ik}f_{kj}}{u_{ij}}\right]}^{2}$$

Here, $X_{ij}$ represents the measured concentration, $u_{ij}$ denotes the estimated uncertainty, n is the number of samples, m is the number of chemical component species, and p is the number of emission sources. The model constrains the results to prevent any sample from having a negative contribution. Using the PMF model allows for the assignment of individual weights to each data point, enabling researchers to adjust the influence of each data point based on the reliability of the measurement.

## 3. Results and Discussion

### 3.1. Concentrations of PM_2.5_ and Its Chemical Components

**Figure 2:**
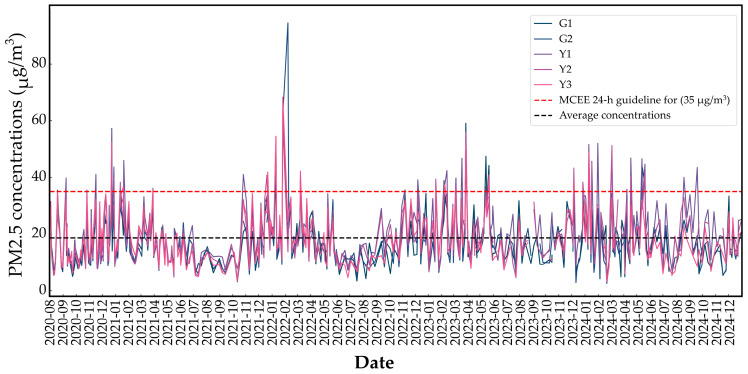
Temporal variations in PM_2.5_ concentrations among the sampling sites.

The temporal distribution of PM_2.5_ concentrations across the five monitoring sites was analyzed over the study period (Figure 2). PM_2.5_ concentrations ranged from 2.55 to 94.52 μg/m^3^, with an average concentration of 18.63 ± 9.71 μg/m^3^. The highest PM_2.5_ levels were observed in spring and winter across all five sites, frequently exceeding the 24 h standard of 35 μg/m^3^ set by the Ministry of Climate, Energy and Environment (MCEE). These results are consistent with findings from Lee (2014), which attributed seasonal variations in PM_2.5_ concentrations to heating-related combustion and the influence of Asian dust [48].

The average concentrations of PM_2.5_, ions, carbonaceous species, and trace elements are presented in Table 2. Among the ionic species, SO_4_^2−^ and NO_3_^−^ exhibited the highest concentrations at 2.93 ± 2.28 μg/m^3^ and 2.48 ± 3.17 μg/m^3^, respectively. Among the trace elements, sulfur (S) had the highest concentration, averaging 1198.20 ± 988.39 ng/m^3^. The concentrations of elemental carbon (EC) and organic carbon (OC) were 0.44 ± 0.33 μg/m^3^ and 3.86 ± 1.92 μg/m^3^, accounting for 2.4% and 20.7% of PM_2.5_ mass, respectively.

According to Choe et al. (2025), EC and OC concentrations in the industrial complexes of Yeosu were reported as 0.53 ± 0.35 μg/m^3^ and 4.09 ± 2.24 μg/m^3^, contributing 2.3% and 17.9% of PM_2.5_ mass, respectively, which is comparable to the findings of this study [49]. In contrast, Kim et al. (2020) reported that EC and OC concentrations in Seoul, the capital and largest metropolitan city of South Korea, were 1.8 μg/m^3^ and 7.3 μg/m^3^, accounting for 5.6% and 22.7% of PM_2.5_ mass, respectively [50]. These values are significantly higher than those observed in this study area. The elevated EC and OC levels in Seoul may primarily be attributed to vehicular emissions [20,21]. In 2021, the average daily passenger and freight traffic volume heading toward Seoul was 3,658,203.2 units, whereas the corresponding volume for Jeollanam-do, the administrative region encompassing Yeosu and Gwangyang, was 1,054,469.5 units [51]. The significantly lower traffic volume in Yeosu and Gwangyang suggests that the lower concentrations and proportions of OC and EC in this study area may be influenced by the reduced impact of vehicular emissions. This finding indicates that, unlike typical urban environments where traffic-related emissions dominate, air pollution in the Yeosu and Gwangyang industrial complexes is more likely influenced by industrial emissions and meteorological factors.

### 3.2. Chemical Characteristics of PM_2.5_ and Its Chemical Components

**Figure 3:**
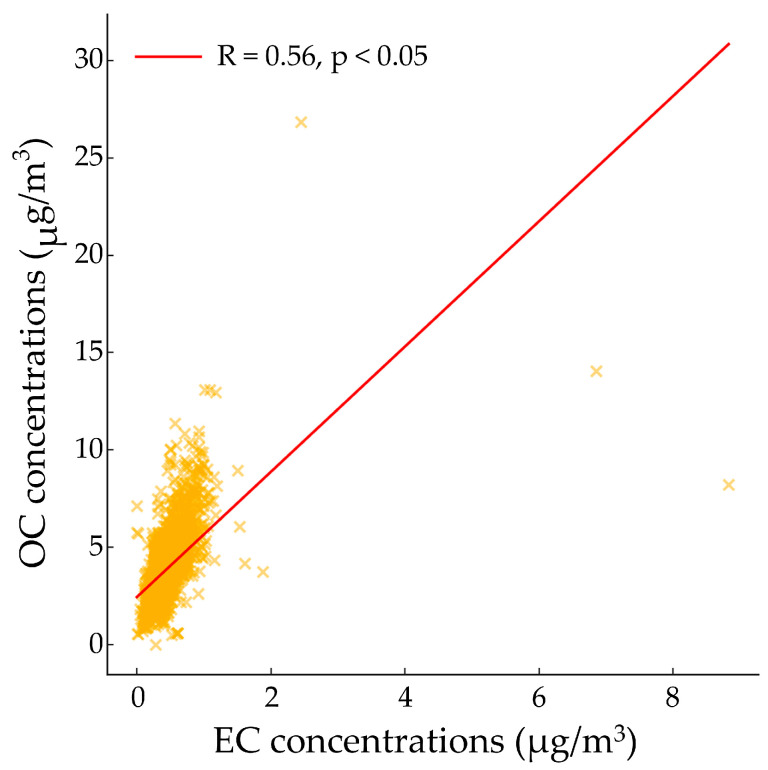
Scatter plot between EC and OC during the sampling period.

**Figure 4:**
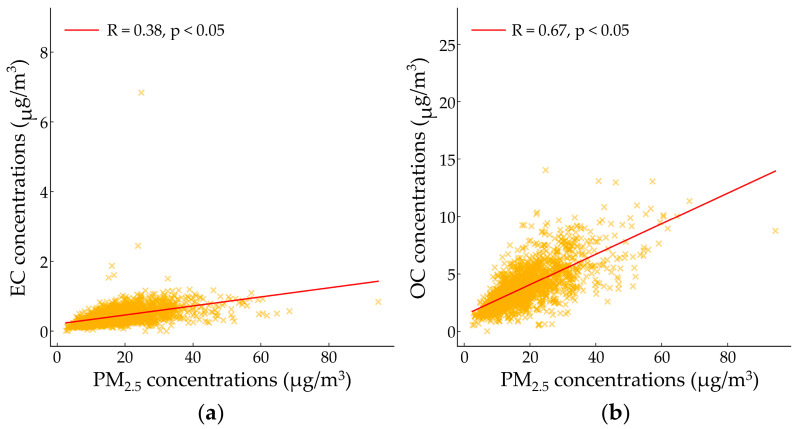
Scatter plot: (**a**) between PM_2.5_ and EC; (**b**) between PM_2.5_ and OC during the sampling period.

The distribution of PM_2.5_ and its chemical components varies depending on emission sources, and each component is considered a fingerprint of its respective source [52]. Correlations among PM_2.5_, carbonaceous species, and ions were analyzed prior to source identification using the PMF model. Figure 3 and Figure 4 present scatter plots of EC versus OC, EC versus PM_2.5_, and OC versus PM_2.5_.

A linear increasing trend was observed between EC and OC; however, the correlation was relatively weak (R = 0.56, *p* < 0.05) (Figure 3). Similarly, both EC and PM_2.5_ and OC and PM_2.5_ exhibited low correlations (R = 0.38, *p* < 0.05; R = 0.67, *p* < 0.05, respectively) (Figure 4a,b) [53].

EC and OC are commonly emitted from vehicular traffic and biomass burning. A strong correlation between EC and OC suggests that these two components share similar emission sources. However, the weak correlations between EC, OC, and PM_2.5_ indicate that secondary aerosol (SA) formation through atmospheric photochemical reactions may significantly contribute to EC and OC levels in the Yeosu and Gwangyang regions [54].

### 3.3. Spatial Variability of PM_2.5_ and Its Chemical Components

The results of the ANOVA analysis for PM_2.5_ chemical components across monitoring sites (Table 2) indicated that all species, except for SO_4_^2−^, NH_4_^+^, EC, OC, V, Mn, Fe, Ni, Zn, As, Mo, Ba, Pb, P, S, and Cr, did not exhibit statistically significant differences (*p* > 0.05). The coefficient of divergence (COD) was calculated (Table 3) to further assess spatial variability among monitoring sites.

Although significant differences in emission sources among the five monitoring sites in Yeosu and Gwangyang were initially expected, the COD values ranged from 0.059 to 0.176. Since these values did not exceed the spatial heterogeneity threshold of 0.3 set in this study, it was determined that the emission sources across monitoring sites were similar. A study by Park et al. (2024) investigated the spatial variability of PM_2.5_ and its chemical components across five monitoring sites in Ansan and Siheung, one of South Korea’s major industrial complexes, and reported COD values ranging from 0.02 to 0.10, indicating identical emission sources [55]. Similarly, Kong et al. (2013) analyzed the spatial similarity of polycyclic aromatic hydrocarbons (PAHs) from six stationary emission sources and reported COD values ranging from 0.10 to 0.29 for five of the sources, suggesting they originated from the same emissions [56].

Based on these findings, it was concluded that the five monitoring sites in this study shared similar emission sources, and source apportionment analysis was conducted under this assumption.

### 3.4. Source Identification in Industrial Complexes

**Figure 5:**
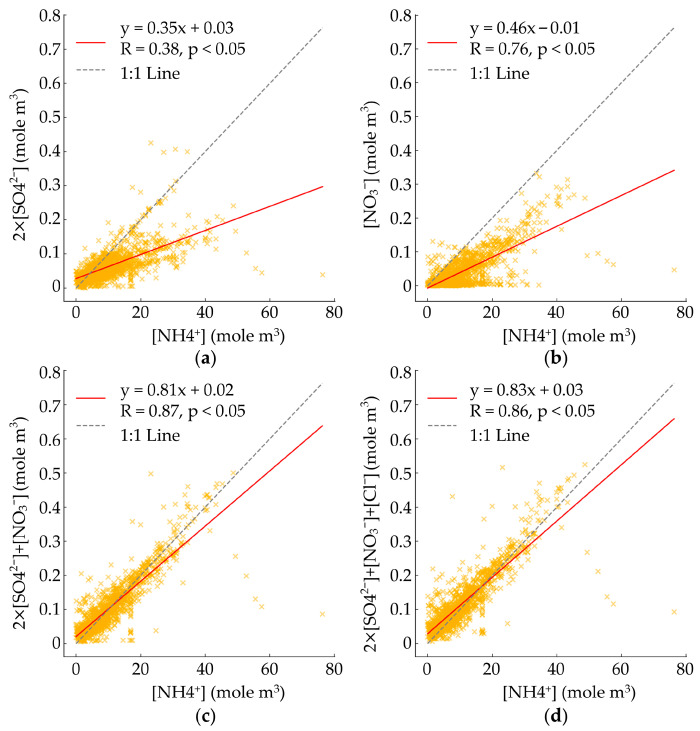
Charge balance: (**a**) between [NH_4_^+^] and 2 × [SO_4_^2−^]; (**b**) between [NH_4_^+^] and [NO_3_^−^]; (**c**) between [NH_4_^+^] and 2 × [SO_4_^2−^] + [NO_3_^−^]; (**d**) between [NH_4_^+^] and 2 × [SO_4_^2−^] + [NO_3_^−^] + [Cl^−^] during study period.

Representative water-soluble inorganic ions (WSII), such as SO_4_^2−^, NO_3_^−^, and NH_4_^+^, have been reported to account for 20–50% of PM_2.5_. Examining their relationships serves as a crucial indicator for the formation and depletion of secondary aerosols [57,58,59]. In this study, the charge balance relationships between [NH_4_^+^] and 2 × [SO_4_^2−^] (R = 0.65, *p* < 0.05), [NH_4_^+^] and [NO_3_^−^] (R = 0.76, *p* < 0.05), [NH_4_^+^] and 2 × [SO_4_^2−^] + [NO_3_^−^] (R = 0.87, *p* < 0.05), and [NH_4_^+^] and 2 × [SO_4_^2−^] + [NO_3_^−^] + [Cl^−^] (R = 0.86, *p* < 0.05) are presented in Figure 5. The regression slopes (Figure 5a,b) indicate that the slope for [NH_4_^+^] and 2 × [SO_4_^2−^] is 0.35, while that for [NH_4_^+^] and [NO_3_^−^] is 0.46. The slope (Figure 5c,d) increases to 0.81 for [NH_4_^+^] and 2 × [SO_4_^2−^] + [NO_3_^−^] and reaches 0.83 for [NH_4_^+^] and 2 × [SO_4_^2−^] + [NO_3_^−^] + [Cl^−^], which is the closest to the 1:1 line. However, all four relationships exhibit slopes lower than 1:1, indicating the dominance of NH_4_^+^. The predominance of NH_4_^+^ in Yeosu and Gwangyang, the study areas, suggests that atmospheric SO_4_^2−^, NO_3_^−^, and Cl^−^ can be neutralized into (NH_4_)_2_SO_4_, NH_4_NO_3_, and NH_4_Cl, respectively.

**Figure 6:**
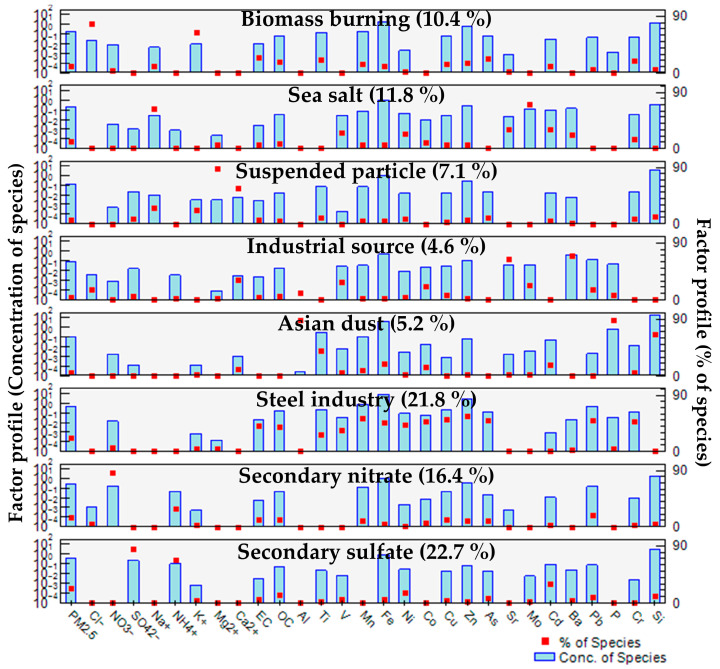
Factor profiles of PMF modeling results of PM_2.5_ and its chemical components.

For source apportionment using PMF modeling, a total of 30 chemical components, including PM_2.5_, and 1747 datasets were utilized. In PMF modeling, the Q value is provided in two forms—$Q_{true}$ and $Q_{robust}$. $Q_{true}$ is calculated using all data points, whereas $Q_{robust}$ accounts for adjusted residuals, making it more reliable under conditions of high uncertainty. As uncertainty increases, these two values converge. The optimal model was selected based on the smallest Q value, where the $Q_{true}$/$Q_{robust}$ ratio was closest to 1. The variables used in PMF modeling are summarized in Table S7. The PMF modeling results identified eight factors, and the temporal variations in their contributions were analyzed (Figure 6 and Figure S2). Classical bootstrap (BS) is a method used to evaluate the uncertainty of PMF results arising from rotational ambiguity and random errors. In the BS procedure, multiple PMF solutions are generated using a series of resampled datasets. Each BS factor is mapped to a corresponding factor from the base run. If no base factor shows a correlation exceeding a predefined threshold with a given BS factor, that factor is defined as unmapped. In this study, 200 BS runs were performed. For each method, all eight resolved factors were mapped with a rate of 100%, indicating that the PMF results obtained in this study are overall stable (Figure S3).

Factor 1 contributed 10.4% of the PM_2.5_ mass fraction. K^+^ and Cl^−^ are well-known indicators of biomass burning and can be emitted from fertilizers such as KCl and NH_4_Cl [60,61]. These species are commonly associated with combustion-related emissions in both residential and agricultural settings. Therefore, Factor 1 was identified as biomass burning.

Factor 2 contributed 11.8% of the PM_2.5_ mass fraction. The high contribution of Na^+^ suggests its origin from marine and sea salt sources [62,63]. This factor represents a typical natural background source in coastal environments. Considering the proximity of the study area to the sea, Factor 2 was identified as sea salt.

Factor 3 contributed 7.1% of the PM_2.5_ mass fraction. Ca^2+^ and Mg^2+^ can originate from resuspended soil particles [24], and Ca^2+^ can also be derived from lime (CaO), a primary component of cement used in construction materials. This factor reflects mixed contributions from natural soil dust and anthropogenic construction activities. Therefore, Factor 3 was identified as suspended particles.

Factor 4 contributed 4.6% of the PM_2.5_ mass fraction. The high contributions of Sr and Ba suggest an origin from industrial activities, including the electronics industry (Ba-Sr related industry) [64,65]. Although this factor accounted for a relatively small fraction of the total PM_2.5_ mass, the enrichment of these elements indicates a distinct industrial emission signature. Consequently, Factor 4 was identified as an industrial source.

Factor 5 contributed 5.2% of the PM_2.5_ mass fraction. Al and Si are well-known crustal elements, while P has been reported to be emitted from bioaerosols such as pollen [66]. The factor contribution analysis over time (Figure S2) demonstrated a higher contribution in spring, reflecting the seasonal characteristics of South Korea. This seasonal pattern is consistent with long-range transported dust events in East Asia. Therefore, Factor 5 was identified as Asian dust.

Factor 6 contributed 21.8% of the PM_2.5_ mass fraction. Trace metal elements, including Ti, V, Mn, Fe, Ni, Co, Cu, Zn, As, Pb, and Cr, showed high contributions. Fe is a dominant metal emitted from the steel industry, while Mn, Cu, Zn, Pb, and Cr can be considered marker elements for steel production emissions [67]. Ti, V, Mn, and Co can also be emitted from metallurgical processes and foundries [68,69,70]. This factor accounted for a substantial contribution of metal-enriched PM_2.5_, highlighting its importance as a source of potentially toxic metals. Given the regional characteristics of large-scale steel industries, Factor 6 was identified as the steel industry.

Factor 7 contributed 16.4% of the PM_2.5_ mass fraction, presenting a high contribution of NO_3_^−^ and a moderate contribution of NH_4_^+^. This composition is indicative of secondary nitrate formation through atmospheric reactions. Factor 8 contributed 22.7% of the PM_2.5_ mass fraction, with strong contributions from SO_4_^2−^ and NH_4_^+^. Secondary aerosols (SA) are primarily composed of (NH_4_)_2_SO_4_ and NH_4_NO_3_, which are derived from gaseous precursors such as NH_3_, SO_2_, and NO_x_ [71]. The significant contributions of SO_4_^2−^, NO_3_^−^, and NH_4_^+^ in these two factors support the presence of secondary aerosols, leading to their identification as secondary nitrate (Factor 7) and secondary sulfate (Factor 8), respectively.

**Figure 7:**
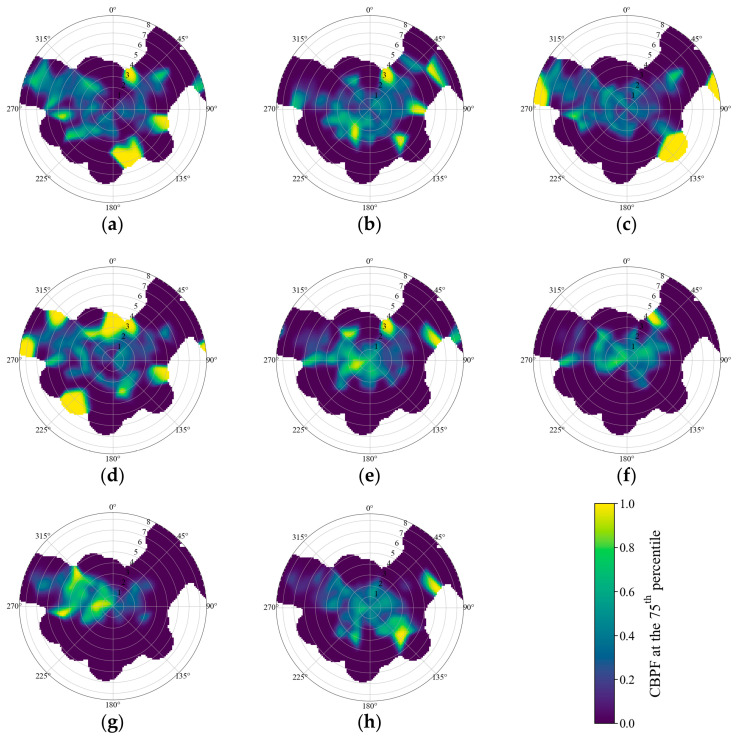
Bivariate polar plots of CBPF modeling results for the 8 factors identified by PMF modeling: (**a**) biomass burning; (**b**) sea salt; (**c**) suspended particle; (**d**) industrial source; (**e**) Asian dust; (**f**) steel industry; (**g**) secondary nitrate; (**h**) secondary sulfate.

To characterize the potential locations and transport patterns of the identified sources, a smoothed Conditional Bivariate Probability Function (CBPF) analysis was conducted for each PMF factor (Figure 7). While the traditional Conditional Probability Function (CPF) primarily identifies source directionality based on wind direction alone, the CBPF approach extends this framework by incorporating wind speed as a radial variable, thereby providing a two-dimensional conditional probability distribution. In this study, high factor contribution events were defined using the 75th percentile of the total contribution for each factor. To enhance the robustness of the spatial distribution and minimize statistical noise in sectors with sparse data, a Gaussian kernel smoothing technique was applied. In the resulting polar plots, high-probability zones located near the center (low wind speed conditions) are generally associated with the influence of local or ground-level sources, whereas probability patterns extending toward the periphery (higher wind speeds) may indicate contributions from elevated emission sources or medium-to-long-range transport. This bivariate approach is particularly effective for disentangling the complex emission characteristics of the Yeosu and Gwangyang industrial complexes, where multiple point and area sources are densely distributed.

Factor 1 (Figure 7a) was characterized by the coupled influence of long-range transport and local emissions. High CBPF probabilities were concentrated in the southeast direction at high wind speeds (>5 m/s), indicating a significant influence of transboundary biomass burning. In contrast, a distinct hotspot was observed in the northeast direction at lower wind speeds (3–4 m/s), likely attributable to local agricultural residue burning or residential heating. These results indicate that biomass-derived aerosols in the study area are governed by both regional atmospheric transport and localized emission sources.

Factor 2 (Figure 7b) was characterized by a distinct spatial distribution associated with maritime influence. Elevated CBPF probabilities were primarily observed in the northeast direction at moderate wind speeds (3–6 m/s), indicating the influence of marine air masses originating from the nearby coastal area. This pattern suggests that sea salt aerosols are transported inland under moderate onshore wind conditions.

Factor 3 (Figure 7c) was characterized by a broad distribution across various wind conditions. High CBPF probabilities were observed in the southeast, northwest, and northeast directions. Hotspots at low to moderate wind speeds (2–4 m/s) suggest contributions from localized sources, such as road dust resuspension or nearby construction activities. In contrast, probability enhancements at higher wind speeds (>6 m/s) in the same directional ranges indicate the potential influence of wind-blown dust or regional atmospheric transport. Overall, suspended particles in the study area appear to be governed by a combination of local mechanical disturbances and regional transport processes.

Factor 4 (Figure 7d) was characterized by pronounced probability enhancements in the north direction. Distinct high-probability signals observed at low wind speeds (<3 m/s) suggest a dominant influence of nearby emission sources located in this direction. This pattern indicates that the observed contributions are mainly driven by local industrial activities or nearby discharge facilities rather than long-range atmospheric transport.

Factor 5 (Figure 7e) was characterized by a broad distribution of high-probability signals in the west to northwest directions. These signals were observed across a wind speed range of (2–6 m/s), indicating the influence of long-range atmospheric transport. This pattern is consistent with the transport of Asian dust particles from distant continental source regions to the sampling site under prevailing westerly and northwesterly wind conditions.

Factor 6 (Figure 7f) was characterized by a highly localized and intense probability enhancement in the northeast direction at moderate wind speeds (3–5 m/s). This directional signal corresponds closely with the location of the Gwangyang Steel Complex, suggesting that emissions associated with iron and steel production processes are a dominant contributor to this factor. The localized nature of the hotspot indicates a strong influence of specific industrial operations rather than broadly distributed sources.

Factor 7 (Figure 7g) was characterized by a relatively diffuse spatial distribution, with enhanced CBPF probabilities concentrated near the center of the plot at low wind speeds (<2 m/s). This pattern implies that nitrate formation and accumulation are favored under stagnant meteorological conditions, where limited atmospheric dispersion and elevated precursor concentrations promote local or near-field secondary aerosol production. Factor 8 (Figure 7h) was characterized by probability enhancements extending toward higher wind speeds (>4 m/s), particularly in the south and southeast directions, showing a distinct pattern compared to nitrate despite also being a secondary aerosol.

However, this study has several limitations. First, the temporal resolution of the measurements was relatively low, as samples were collected once or twice per week, which may limit the ability to fully capture short-term variability in PM_2.5_ concentrations and chemical composition. Second, the variation in sampling frequency between these intervals indicates that the interpretation of PMF and CBPF results require careful consideration. Nevertheless, the long-term dataset and the large number of samples collected over multiple years provide a comprehensive foundation for identifying dominant sources and overall PM_2.5_ characteristics in the study area, offering fundamental information for future toxicology, exposure, and health risk assessment studies.

## 4. Conclusions

In this study, PM_2.5_ concentrations ranged from 2.55 to 94.52 μg/m^3^, with an average concentration of 18.63 ± 9.71 μg/m^3^. The time-series analysis of concentration distribution indicated that high concentrations were observed during winter and spring throughout the study period. The coefficient of divergence (COD) was calculated to assess spatial variability, revealing no significant differences in emission sources among the five measurement sites within the study area.

The relationship between EC, OC, and PM_2.5_ during the study period presented a low correlation between EC and OC (R = 0.56), suggesting that the emitted EC and OC may indicate conditions favorable for the dominance of secondary aerosols. The charge balance analysis of [NH_4_^+^] with [SO_4_^2−^], [NO_3_^−^], and [Cl^−^] showed that all relationships exhibited slopes lower than the 1:1 line, indicating that NH_4_^+^ neutralizes SO_4_^2−^, NO_3_^−^, and Cl^−^ to form (NH_4_)_2_SO_4_, NH_4_NO_3_, and NH_4_Cl, respectively.

PMF modeling for source identification identified eight factors, revealing that the major emission sources in the atmosphere of Yeosu and Gwangyang were biomass burning (10.4%), sea salt (11.8%), suspended particles (7.1%), industrial sources (4.6%), Asian dust (5.2%), steel industry (21.8%), secondary nitrate (16.4%), and secondary sulfate (22.7%).

This study conducted long-term atmospheric measurements and analyses in Yeosu and Gwangyang to investigate the chemical characteristics and emission sources of PM_2.5_ and its components. Understanding the distribution characteristics and source-specific contributions of PM_2.5_, particularly those associated with toxic and potentially hazardous trace elements, can contribute to the development of targeted mitigation strategies for localized pollution and the establishment of effective emission control policies. Furthermore, the findings of this study provide essential baseline data that can be directly utilized in future toxicology and quantitative health risk assessment studies focusing on PM_2.5_ exposure in industrial regions.

## Figures and Tables

**Table 1:** Description of the sampling sites in this study.

Area	Site Designation	Coordinates	Nearest Emission Source	Distance from Sampling Site
Gwangyang	G1	34°56′35.76″ N, 127°44′58.45″ E	Gwangyang Steel Complex	900 m
	G2	34°56′14.54″ N, 127°40′45.00″ E	Gwangyang Seonghwang General Industrial Complex	2100 m
Yeosu	Y1	34°52′55.08″ N, 127°34′43.40″ E	Yulchon General Industrial Complex	1900 m
	Y2	34°47′54.01″ N, 127°37′54.65″ E	Yeosu National Industrial Complex	4800 m
	Y3	34°50′02.87″ N, 127°44′05.85″ E	Yeosu National Industrial Complex	5500 m

**Table 2:** The average concentrations of PM_2.5_, ions, carbon, and trace elements in industrial complexes (μg/m^3^ for PM_2.5_, ions, and carbon; ng/m^3^ for trace elements).

Chemical Components	*N*	Average	Median	Range	*p*-Value ^a^
PM_2.5_	1767	18.63 ± 9.71	16.45	2.55–94.52	0.000 *
Cl^−^	1649	0.36 ± 0.49	0.24	0.01–12.44	0.274
NO_3_^−^	1542	2.48 ± 3.17	1.07	0.01–20.61	0.429
SO_4_^2−^	1698	2.93 ± 2.28	2.43	0.01–29.15	0.000 *
Na^+^	1639	0.47 ± 0.44	0.41	0.01–8.69	0.746
NH_4_^+^	1673	1.76 ± 1.48	1.33	0.01–13.76	0.011 *
K^+^	1545	0.19 ± 0.19	0.16	0.01–3.58	0.333
Mg^2+^	811	0.10 ± 0.08	0.09	0.01–0.52	0.878
Ca^2+^	1199	0.14 ± 0.25	0.11	0.01–7.86	0.419
EC	1813	0.44 ± 0.33	0.39	0.01–8.83	0.039 *
OC	1813	3.86 ± 1.92	3.5	0.02–26.83	0.000 *
Al	291	406.2 ± 564.02	227.27	0.67–3443.03	0.904
Ti	1599	9.87 ± 13.85	6.59	0.05–161.50	0.161
V	1567	1.46 ± 1.19	1.15	0.05–8.31	0.000 *
Mn	1765	13.65 ± 13.04	10.72	0.10–291.28	0.000 *
Fe	1767	189.76 ± 169.95	147.8	5.58–1758.69	0.000 *
Ni	1735	2.36 ± 10.82	1.54	0.05–392.54	0.000 *
Co	1611	1.16 ± 0.96	0.95	0.03–9.28	0.000 *
Cu	1764	3.97 ± 3.38	3.24	0.05–58.91	0.265
Zn	1767	43.31 ± 29.85	36.78	0.85–309.33	0.000 *
As	1662	2.94 ± 2.47	2.29	0.05–16.20	0.000 *
Sr	1016	1.41 ± 1.83	1.05	0.03–46.62	0.258
Mo	1049	3.60 ± 4.66	2.34	0.02–72.41	0.000 *
Cd	1154	4.15 ± 4.11	2.94	0.02–40.20	0.680
Ba	1117	11.44 ± 10.88	8.5	0.02–104.36	0.007 *
Pb	1708	9.69 ± 12.78	8.32	0.05–454.92	0.025 *
P	1234	9.82 ± 10.58	6.65	0.03–84.44	0.000 *
S	1754	1198.20 ± 988.39	909.72	2.45–8539.38	0.000 *
Cr	1735	2.76 ± 5.81	2.11	0.05–196.00	0.000 *
Si	1745	355.58 ± 592.68	209.9	0.79–8336.61	0.486

^a^ ANOVA results among the sites, * significantly different (*p* < 0.05).

**Table 3:** Coefficient of divergence among the sampling sites.

	G1	G2	Y1	Y2	Y3
G1	0.000	0.146	0.121	0.131	0.176
G2		0.000	0.072	0.059	0.103
Y1			0.000	0.059	0.124
Y2				0.000	0.095
Y3					0.000

## Data Availability

The data used in this study are available upon reasonable request, subject to approval by the Ministry of Climate, Energy and Environment (MCEE).

## Supplementary Materials

The following supporting information can be downloaded at https://www.mdpi.com/article/10.3390/toxics14020111/s1, Figure S1: Seasonal wind roses at the study site: (a) spring; (b) summer; (c) autumn; (d) winter; Figure S2: Time-series contribution of each table results of PMF modeling; Figure S3: Bootstrap variability of PMF factor profiles; Table S1: Seasonal summary of meteorological factors during the study period; Table S2: The specification of the measurement device used in this study; Table S3: Ion chromatography analysis equipment and conditions; Table S4: Flame ionization detector analysis equipment and conditions; Table S5: Energy dispersive X-ray fluorescence analyzer and conditions; Table S6: QA/QC results of the chemical components analyzed in this study; Table S7: Variables used in PMF modeling.

## Abbreviations

The following abbreviations are used in this manuscript:

PM	Particulate matter
WHO	World health organization
PM_2.5_	Particulate matter with an aerodynamic diameter less than 2.5 μm
VOCs	Volatile organic compounds
HMs	Heavy metals
WHIIs	Water-soluble organic ions
OC	Organic carbon
EC	Elemental carbon
PMF	Positive matrix factorization
ED-SRF	Energy-dispersive X-ray fluorescence
QA/QC	Quality assurance and quality control
MDL	Minimum detection level
RSD	Relative standard deviation
ANOVA	Analysis of variance
COD	Coefficient of divergence
PAHs	Polycyclic aromatic hydrocarbon
CBPF	Conditional bivariate probability function
CPF	Conditional probability function
