# Supplementary material for: Chemical Characteristics and Source Identification of PM2.5 in Industrial Complexes, Korea
Source: Toxics. 2026 Jan 23;14(2):111. doi: 10.3390/toxics14020111 (PMC12945190; doi:10.3390/toxics14020111)
Supplement: Supplementary file 1 [file toxics-14-00111-s001.zip › Table S1.pdf]

Table S1. Seasonal summary of meteorological factors during study period.

Factors	Unit	Spring	Summer	Autumn	Winter
Temperature	℃	15.05 ± 5.39	25.99 ± 3.39	17.87 ± 6.07	4.50 ± 4.62
Relative humidity	%	59.98 ± 21.55	78.25 ± 14.57	66.65 ± 17.20	56.07 ± 17.69
Wind speed	m/s	3.34 ± 2.29	3.10 ± 1.65	3.48 ± 1.99	4.49 ± 2.45
Precipitation	mm	0.18 ± 1.14	0.38 ± 2.36	0.17 ± 1.62	0.05 ± 0.48
Station pressure	hPa	1006.54 ± 5.81	999.0 ± 5.67	1009.29 ± 5.67	1014.32 ± 4.67
Sea-level pressure	hPa	1015.89 ± 5.96	1007.91 ± 4.10	1018.53 ± 5.85	1024.15 ± 4.79
