# Supplementary material for: Chemical Characteristics and Source Identification of PM2.5 in Industrial Complexes, Korea
Source: Toxics. 2026 Jan 23;14(2):111. doi: 10.3390/toxics14020111 (PMC12945190; doi:10.3390/toxics14020111)
Supplement: Supplementary file 1 [file toxics-14-00111-s001.zip › Table S2.pdf]

Table S2. The specification of the measurement device used in this study.

Specification	
Device name	PMS-204(APM Co., Ltd, Korea)
Substance	$PM_{2.5}$
Dimensions	366(W)*465(H)*250(D) mm, 15 kg
Measurement method	Gravimetrical method
Filter	Teflon (PTFE), Quartz
Operating temperature	-30~50℃
Internal temperature	±5℃
Power	110/220 VAC 50–60Hz
Flow rate (Flow control accuracy)	0~20L/min (±1.5% @ F.S)
Memory	USB Memory 4GB
