# Supplementary material for: Chemical Characteristics and Source Identification of PM2.5 in Industrial Complexes, Korea
Source: Toxics. 2026 Jan 23;14(2):111. doi: 10.3390/toxics14020111 (PMC12945190; doi:10.3390/toxics14020111)
Supplement: Supplementary file 1 [file toxics-14-00111-s001.zip › Table S3.pdf]

Table S3. Ion chromatography analysis equipment and conditions.

Item	Conditions	
	**Anion**	**Cation**
Separate column	AS-14	CS-12A
Guard column	AG-14	CG-12A
Suppressor	ADRS 4mm	CERS 2 mm
Detector	CD 20	CD 20
Flow rate (LPM)	0.5	0.3
