# Supplementary material for: Chemical Characteristics and Source Identification of PM2.5 in Industrial Complexes, Korea
Source: Toxics. 2026 Jan 23;14(2):111. doi: 10.3390/toxics14020111 (PMC12945190; doi:10.3390/toxics14020111)
Supplement: Supplementary file 1 [file toxics-14-00111-s001.zip › Table S4.pdf]

Table S4. Flame ionization detector analysis equipment and conditions.

**Conditions**	
Detected method	Flame ionization detector
Measuring method	NIOSH 5040, EPA STN Method, User-designed temperature protocols
Analytical techniques	EVOLVED GAS ANALYSIS by thermal-optical analyzer
Analytical components	EC, OC, Carbonate Carbon
Measuring range	OC (1 to 105 ㎍ per filter portion)
Total precision	0.085 at 23 ㎍/㎥
Precision	0.19 at 1 ㎍/C, 0.01 at 10 to 72 ㎍/C
Accuracy	±16.7% at 23 ㎍/㎥
Expected load	0.3 ㎍ per filter portion
Operating characteristics	1) Minimum quantifiable total OC-0.1ug/C<br>2) Minimum quantifiable total EC-0.1ug/C<br>3) Maximum instrument blank contribution-0.1ugC
