# Supplementary material for: Chemical Characteristics and Source Identification of PM2.5 in Industrial Complexes, Korea
Source: Toxics. 2026 Jan 23;14(2):111. doi: 10.3390/toxics14020111 (PMC12945190; doi:10.3390/toxics14020111)
Supplement: Supplementary file 1 [file toxics-14-00111-s001.zip › Table S5.pdf]

Table S5. Energy dispersive X-ray fluorescence analyzer and conditions.

Conditions	
X-Ray optics	-
Composed of 80° of the X-ray tube and detector	Yes
Distance between SDD detector and sample	less than 15mm
Distance between X-Ray tube target and sample	less than 40mm
X-ray tube	-
End-window type with Rh target	End window Ag target, 15W
50um Be window, air cooled	Yes
X-Ray generator	-
Range of voltage	4 ~ 50kV
Range of current	0 ~ 3.0mA
Activated area of 30 ㎟, 20 ㎟ collimated	Yes
Resolution	140eV@Mn Ka 100kcps
Maximum count rate	1.5Mcps
Window	8um (0.315mil) Beryllium
