# Supplementary material for: Chemical Characteristics and Source Identification of PM2.5 in Industrial Complexes, Korea
Source: Toxics. 2026 Jan 23;14(2):111. doi: 10.3390/toxics14020111 (PMC12945190; doi:10.3390/toxics14020111)
Supplement: Supplementary file 1 [file toxics-14-00111-s001.zip › Table S6.pdf]

Table S6. QA/QC results of the chemical components analyzed in this study.

Chemical Components	Method detection limits	Relative standard deviation (%)
Cl^-	0.036	0.10
NO_3^-	0.008	0.10
SO_4^{2-}	0.015	0.10
Na^+	0.016	0.12
NH_4^+	0.011	0.11
K^+	0.006	0.18
Mg^{2+}	0.005	0.11
Ca^{2+}	0.004	0.12
EC	-	-
OC	0.31	1.1
Al	59.28	0.59
Ti	5.64	0.51
V	6.15	0.53
Mn	0.47	0.09
Fe	2.79	0.07
Ni	1.93	0.34
Co	2.47	0.10
Cu	1.42	0.06
Zn	1.70	0.23
As	1.43	0.22
Sr	0.49	0.07
Mo	3.66	0.48
Cd	14.82	1.44
Ba	3.86	0.12
Pb	2.66	0.04
P	1.84	0.26
S	40.68	0.31
Cr	0.47	0.04
Si	6.20	0.39
