# Supplementary material for: Chemical Characteristics and Source Identification of PM2.5 in Industrial Complexes, Korea
Source: Toxics. 2026 Jan 23;14(2):111. doi: 10.3390/toxics14020111 (PMC12945190; doi:10.3390/toxics14020111)
Supplement: Supplementary file 1 [file toxics-14-00111-s001.zip › Table S7.pdf]

Table S7. Variables used in PMF modeling.

Chemical components	S/N	Category	Slope	SE	R^{2*}
Cl^-	9.00	Strong	0.87	3.87	0.80
NO_3^-	5.93	Strong	0.40	0.16	0.48
SO_4^{2-}	7.18	Strong	1.00	0.64	0.96
Na^+	8.27	Strong	0.78	0.80	0.82
NH_4^+	7.70	Strong	0.43	0.17	0.46
K^+	8.40	Strong	0.83	0.55	0.83
Mg^{2+}	5.95	Strong	0.60	0.07	0.61
Ca^{2+}	3.42	Strong	0.54	0.03	0.54
EC	5.55	Strong	0.42	0.06	0.41
OC	2.35	Strong	0.56	0.14	0.46
Al	8.39	Strong	0.78	0.88	0.71
Ti	1.34	Weak	0.00	0.04	0.61
V	3.57	Strong	0.47	3.22	0.75
Mn	1.82	Weak	0.31	0.49	0.28
Fe	8.65	Strong	0.56	3.52	0.71
Ni	8.98	Strong	0.70	33.03	0.90
Co	3.61	Strong	0.05	0.78	0.09
Cu	2.30	Strong	0.74	0.29	0.81
Zn	6.25	Strong	0.58	1.11	0.62
As	8.83	Strong	0.75	9.01	0.80
Sr	5.02	Strong	0.25	0.97	0.23
Mo	3.79	Strong	0.39	0.42	0.49
Cd	2.86	Strong	0.30	1.01	0.32
Ba	1.86	Weak	-0.03	1.52	0.00
Pb	4.34	Strong	0.63	3.58	0.65
P	7.05	Strong	0.46	3.63	0.48
S	5.25	Strong	1.29	7.01	0.68
Cr	8.86	Bad	0.16	0.90	0.19
Si	7.47	Strong	0.69	85.25	0.95

*Regression diagnostics.
