# Supplementary figures and images for: Chemical Characteristics and Source Identification of PM2.5 in Industrial Complexes, Korea
Source: Toxics. 2026 Jan 23;14(2):111. doi: 10.3390/toxics14020111 (PMC12945190; doi:10.3390/toxics14020111)

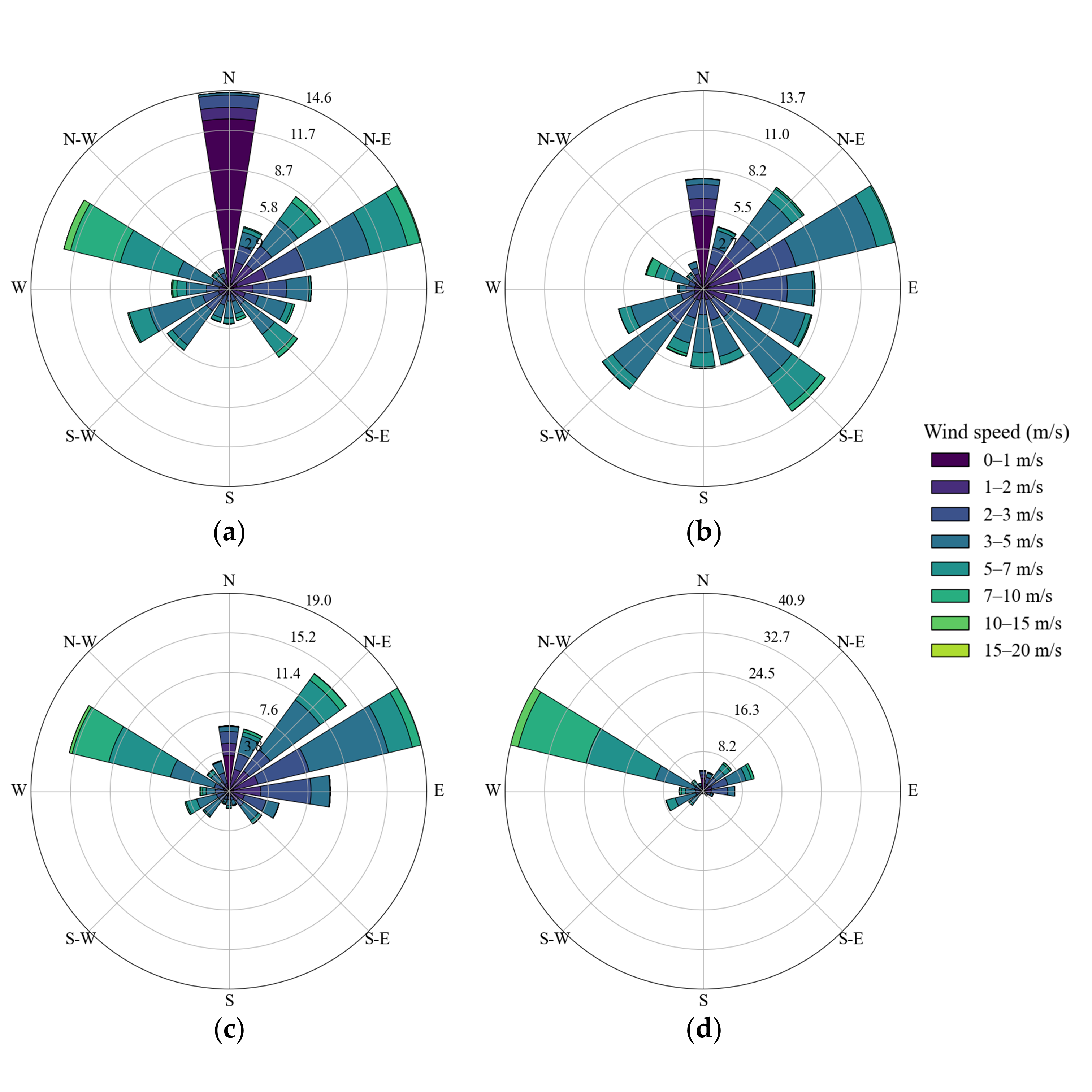

Supplement: Supplementary file 1 [file toxics-14-00111-s001.zip › Figure S1.png]

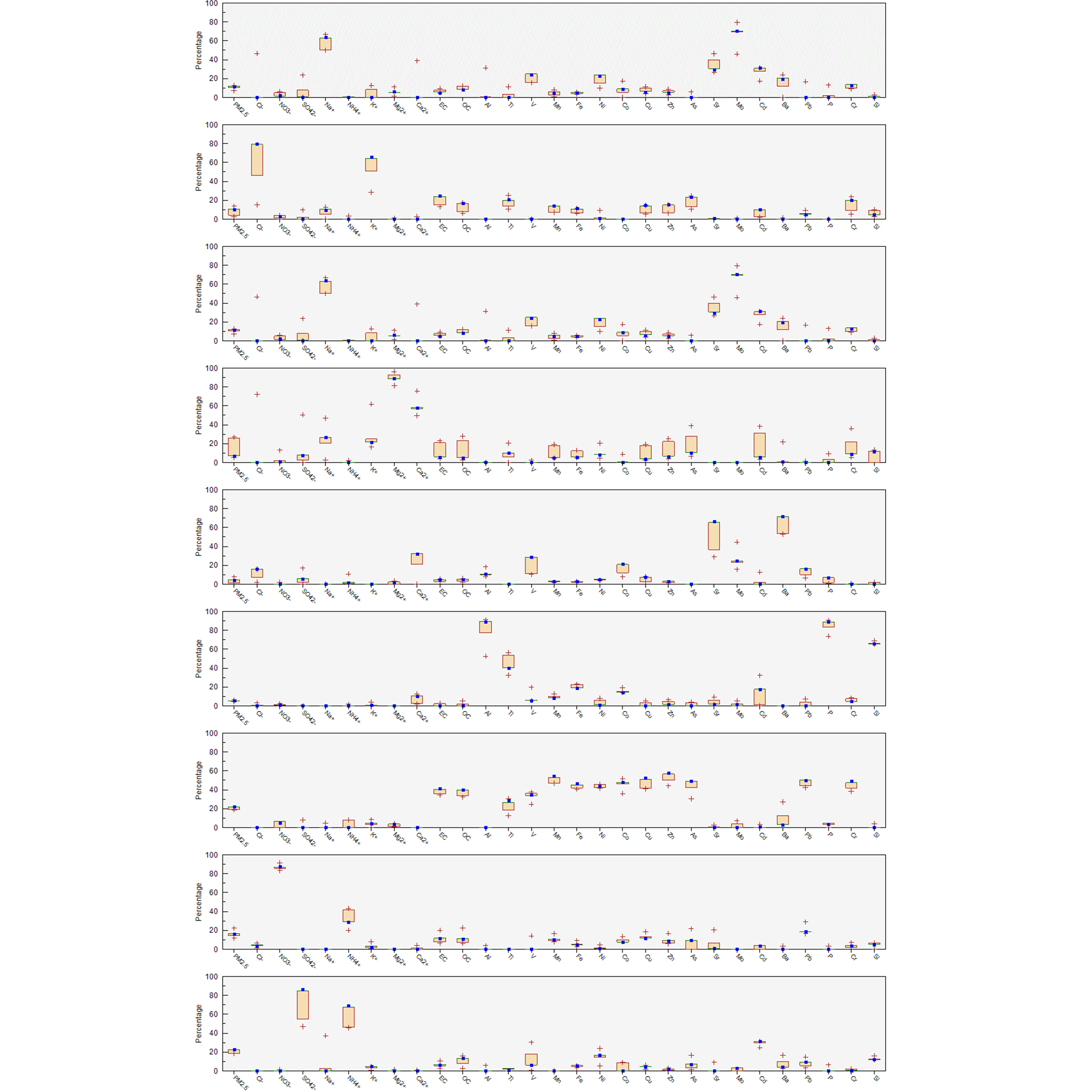

Supplement: Supplementary file 1 [file toxics-14-00111-s001.zip › Figure S3.png]
